# Long non-coding RNA AFAP1-AS1 facilitates the growth and invasiveness of oral squamous cell carcinoma by regulating the miR-145/HOXA1 axis

**DOI:** 10.3892/or.2022.8306

**Published:** 2022-03-21

**Authors:** Minghe Li, Dongsheng Yu, Zhihong Li, Cong Zhao, Chang Su, Jun Ning

Oncol Rep 45: 1094–1104, 2021; DOI: 10.3892/or.2020.7908

Following the publication of the above article, an interested reader drew the authors’ attention to the fact that certain features shown in Fig. 6B, illustrating the tumors extracted from the animal *in vivo* experiments, were strikingly similar to images that had appeared in other papers by different authors published at around the same time. The authors conceded that the *in vivo* experiments reported in this study had been performed by a third party. Therefore, in the interests of preserving accuracy in the scientific record, the authors requested that this paper be retracted from the Journal.

The Editor is in agreement that the paper should be retracted. All authors agree with the retraction of this article, and the Editor apologizes to the readership for any inconvenience caused.

